# Nuclear control of lung cancer cells migration, invasion and bioenergetics by eukaryotic translation initiation factor 3F

**DOI:** 10.1038/s41388-019-1009-x

**Published:** 2019-09-16

**Authors:** Pauline Esteves, Laetitia Dard, Aurélia Brillac, Christophe Hubert, Saharnaz Sarlak, Benoît Rousseau, Elodie Dumon, Julien Izotte, Marc Bonneu, Didier Lacombe, Jean-William Dupuy, Nivea Amoedo, Rodrigue Rossignol

**Affiliations:** 1 Bordeaux University, 146 rue Léo Saignat, 33000 Bordeaux, France; 2 INSERM U1211, 33000 Bordeaux, France; 3 Transgenic Animal Facility A2, University of Bordeaux, 33000 Bordeaux, France; 4 Functional Genomics Center (CGFB), Proteomics Facility, 146 Rue Léo Saignat, 33076 Bordeaux, France; 5 Bordeaux-INP, Avenue des Facultés, 33405 Talence Cedex, France; 6 CELLOMET, Functional Genomics Center (CGFB), 146 rue Léo Saignat, 33000 Bordeaux, France

**Keywords:** Biochemistry, Cancer

## Abstract

The basic understanding of the biological effects of eukaryotic translation initiation factors (EIFs) remains incomplete, notably for their roles independent of protein translation. Different EIFs exhibit nuclear localization and DNA-related functions have been proposed, but the understanding of EIFs novel functions beyond protein translation lacks of integrative analyses between the genomic and the proteomic levels. Here, the noncanonical function of EIF3F was studied in human lung adenocarcinoma by combining methods that revealed both the protein–protein and the protein–DNA interactions of this factor. We discovered that EIF3F promotes cell metastasis in vivo. The underpinning molecular mechanisms involved the regulation of a cluster of 34 metastasis-promoting genes including Snail2, as revealed by proteomics combined with immuno-affinity purification of EIF3F and ChIP-seq/Q-PCR analyses. The interaction between EIF3F and signal transducer and activator of transcription 3 (STAT3) controlled the EIF3F-mediated increase in Snail2 expression and cellular invasion, which were specifically abrogated using the STAT3 inhibitor Nifuroxazide or knockdown approaches. Furthermore, EIF3F overexpression reprogrammed energy metabolism through the activation of AMP-activated protein kinase and the stimulation of oxidative phosphorylation. Our findings demonstrate the role of EIF3F in the molecular control of cell migration, invasion, bioenergetics, and metastasis. The discovery of a role for EIF3F–STAT3 interaction in the genetic control of cell migration and metastasis in human lung adenocarcinoma could lead to the development of diagnosis and therapeutic strategies.

## Introduction

The molecular mechanisms of tumorigenesis are classified as hallmarks as diverse as resistance to cell death, activation of invasion, and metastasis or deregulation of cellular energetics [1]. Acquisition of these malignant properties requires numerous alterations in cell signaling at the level of gene transcription, protein translation and posttranslational modifications, as coordinated by various oncogenes, tumor suppressors, transcription factors, and the tumor microenvironment. Recently, the eukaryotic initiation factors (EIFs) were proposed as emerging players in the acquisition of malignant tumor properties, but the underpinning molecular mechanisms remain unclear [2]. EIFs are commonly involved in the initiation of protein translation, which is a highly regulated rate-limiting step and is dependent on the coordinated actions of several EIFs [3]. Aberrant expression of different EIFs was found in various human tumors, and cancer cell biology studies revealed their respective roles in cell transformation and tumor heterogeneity [2, 4]. Different biological effects were reported following the ectopic overexpression of specific EIFs in the spontaneously immortalized murine NIH3T3 cell line, ranging from the stimulation of cell proliferation, as observed for EIF4E, EIF4B, or EIF5A2, to the control of apoptosis, as observed for EIF5A1. Mechanistically, it is proposed that EIFs can specifically regulate subsets of mRNAs based on elements found in their sequence, suggesting that defined modules of genes are activated in tumors with aberrant expression of the corresponding EIFs. As a result, some EIFs were considered to be of major interest for the development of novel anti-cancer therapeutic strategies [2]. Therefore, a better knowledge of the link between the aberrant expression of specific EIFs and cancer cell biology is required to develop adapted therapeutic approaches.

The basic understanding of the biological effects of EIFs also remains incomplete, notably for their roles independent of protein translation. For instance, different EIFs such as EIF4E, EIF5A, or EIF3F exhibit nuclear localization and proposed DNA-related functions [5–7]. In particular, biochemical studies revealed an intriguing ability for EIF3F to bind to several nuclear proteins such as Ataxin 1, Cyclin A1 or Notch (see full list in Supplementary information Table S1). These proteins are involved in the regulation of the cell cycle (GO:0007049), nuclear division (GO:0000280) and organelle organization (GO:0006996), as well as transcription-factor binding (GO:0001025), suggesting a possible nuclear role for EIF3F in the regulation of gene expression. Accordingly, Gutiérrez-Fernández et al. [8] proposed that EIF3F has the ability to interact with different proteins located in the nucleus due to the presence of a Mpr1/Pad N-terminal motif that mediates protein–protein interactions and that promotes the assembly of large complexes. Therefore, we hypothesized that the participation of EIF3F in the cancer cells malignancy could involve unknown nuclear mechanisms that require partnering with unidentified proteins including genetic regulators of carcinogenesis. The distribution of EIF3F expression in lung adenocarcinoma (LUAD) remains unclear and large-cohort analyses, as proposed by The Cancer Genome Atlas (Genomic Data Common (GDC)-TCGA [9]), are required to study the distribution of EIF3F expression in the disease.

In our study, we observed that ectopic overexpression of EIF3F in human lung cancer cells alters cell proliferation and bioenergetics but also promotes metastasis in vivo. Using proteomics combined with protein–protein interaction studies and ChIP-seq analysis, we identified a set of 34 genes that were specifically bound to and regulated by EIF3F and that were involved in the control of energy metabolism, cell spreading, and metastasis progression. We discovered that the genetic control of metastasis by EIF3F requires the transcription factor STAT3. Therefore, our findings identify a novel EIF3F–STAT3 axis involved in the promotion of metastasis.

## Results

### Ectopic overexpression of EIF3F alters human lung cancer cell proliferation

**Fig. 1:**
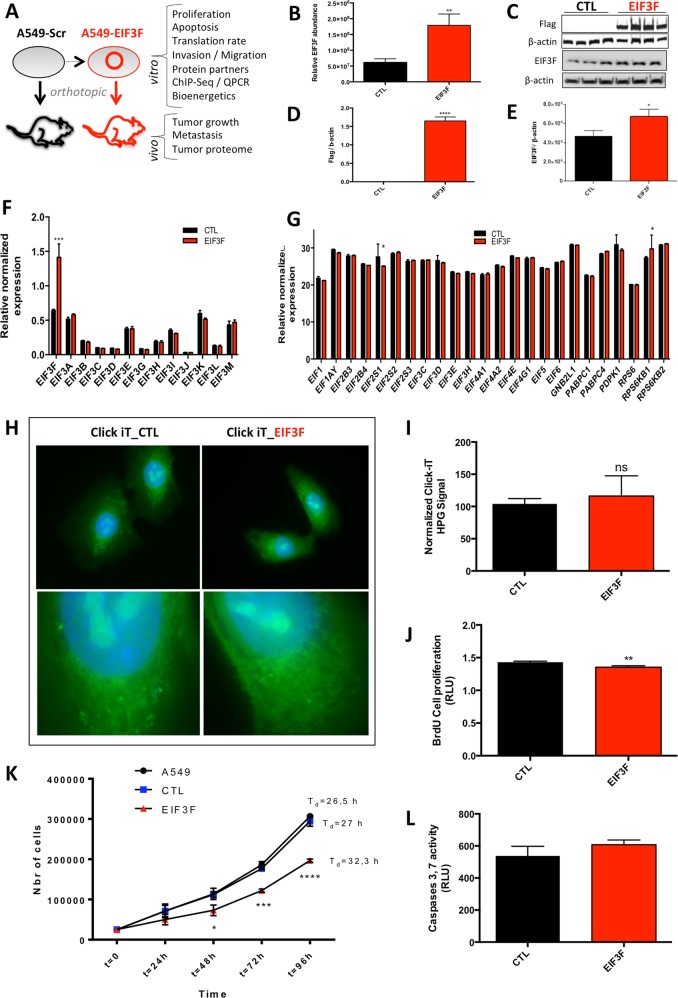
EIF3F overexpression alters A549 human lung cancer cells proliferation. **a** Study workflow including in vitro and in vivo analyses of A549 cells overexpressing EIF3F. **b** Verification of the ectopic expression of EIF3F in A549 human lung adenocarcinoma cells using quantitative proteomics (*N* = 3) and **c**–**e** immunoblot analysis of the M2-FLAG and EIF3F protein expression level in control and EIF3F cells. β-actin was used as loading control for EIF3F expression quantification (*N* = 3 and *N* = 4, respectively). **f** EIF3 complex subunits expression was determined by qPCR (*N* = 3). **g** Expression of PIC (pre initiation complex) components was analyzed using qPCR (*N* = 3). **h** The global rate of protein synthesis was assessed by immunostaining of HPG Alexa Fluor® 488 (*N* = 10) and **i** corresponding quantification. L-homopropargylglycine (HPG) is an amino acid analog of methionine containing an alkyne moiety and Alexa Fluor® 488. The HPG is fed to cultured cells and incorporated into proteins during active protein synthesis. **j** Cell proliferation rate was determined using the BrdU incorporation assay (*N* = 12). **k** Growth curves of A549, CTL-A549, and EIF3F-A549 cells were obtained by performing cell enumeration studies (*N* = 6). **l** Apoptosis was investigated by measuring caspases 3 and 7 activities using a fluorescent substrate (*N* = 4). All data were expressed as mean ± SEM from ‘n’ independent cell cultures. *P *<* 0.05, **P *<* 0.01, ***P *<* 0.001

To study the link between human EIF3F overexpression and neoplasia, we generated EIF3F-A549 LUAD cancer cells that overexpressed a flagged-EIF3F ectopic subunit of the human EIF3 complex (Fig. 1a). Quantitative label-free proteomic analysis of these cells showed a significant fourfold increase in EIF3F protein content (Fig. 1b), and western blot experiments confirmed these findings (Fig. 1c–e). No change was observed in the expression of the other 12 subunits of the EIF3 complex at the mRNA level (Fig. 1f), indicating that the genetic modulation of EIF3F expression was specific. Likewise, we observed no change, or very little change, in the expression of the 24 components of the entire translation pre-initiation complex (PIC), indicating that EIF3F did not alter the global expression of the genes involved in the machinery responsible for initiation of protein translation (Fig. 1g). These findings were in agreement with the functional evaluation of the global rate of protein translation in EIF3F-A549 cells, compared to that in control cells expressing the empty plasmid (Fig. 1h, i). However, the ectopic overexpression of EIF3F altered the A549 cell proliferation rate, as assessed by BrDU incorporation studies (Fig. 1j) or cell enumeration experiments and calculation of the doubling time (Fig. 1k). Moreover, the measurement of caspase 3 and caspase 7 activities revealed no change in the activation of apoptosis in EIF3F-A549 cancer cells (Fig. 1l). These findings suggested a tumor suppressive role for EIF3F in controlling the proliferation of human lung cancer cells, as observed in a previous study performed on melanoma and pancreatic cells [10]. We also observed this tumor suppressive effect of EIF3F overexpression in H1975, HeLa, and H460 human cancer cell lines (Fig. S1).

### EIF3F overexpression in lung cancer cells promotes hepatic metastasis in vivo

**Fig. 2:**
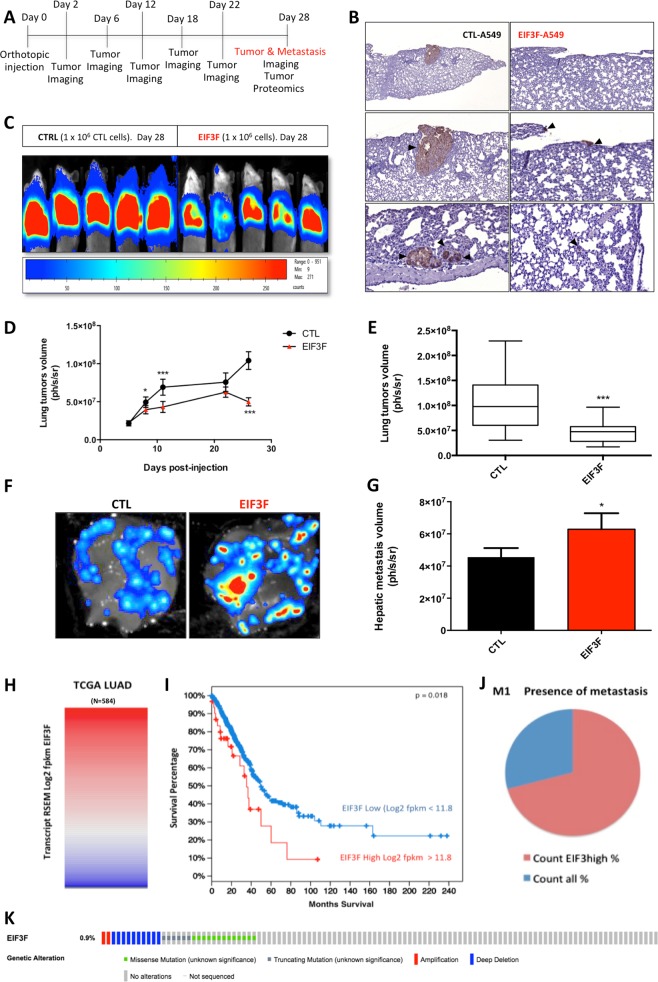
EIF3F overexpression alters lung tumor growth in vivo but promotes hepatic metastasis. **a** Protocol used for mice orthotopic human lung tumors generation and tumor imaging and sampling. **b** Immunostaining of A549 human cells in the lung parenchyma using anti-HLA antibodies allowed verifying the presence of human orthotopic tumor in NSG-mice lung (4 weeks time point). Observation was performed on control mice that received no cell injection and on mice injected with EIF3F-A549 human cancer cells. The lung tissue was stained with HES staining. **c** in vivo determination of the volume of luciferase-expressing mice orthotopic human lung tumors in NSG mice (4 weeks time point). Results are expressed as number of photons per second per steradian. **d** CTL and EIF3F lung tumors volume follow-up during the course of the experiment (20 mice per group). **e** Lung tumors volume after 28 days of growth determined in vivo using bioluminescence imaging (*N* = 20). **f** Identification and quantification of hepatic metastasis in the two groups of mice. **g** Quantification of the volume of hepatic metastasis was performed ex vivo (*N* = 20). **h** EIF3F gene expression was analyzed in silico on a cohort of 584 lung adenocarcinoma tumors (RNAseqV2 data from the TCGA LUAD cohort). EIF3F high and EIF3F low populations were determined by setting the cut-off for EIF3F expression at 11.8, based on calculations using the Cbioportal bioinformatic platform. This cut-off value of 11.8 segregates two groups of patients with a significant difference in survival (*p* = 0.018). The group of patients with EIF3F expression >11.8 accounted for 10% of the total population. **i** Patients survival curves were obtained for the two subgroups stratified by EIF3F expression (high or low; as obtained from **h**). **j** The presence of metastasis (M1 population) was compared in EIF3F high and EIF3F low LUAD patients (TCGA data). **k** Genetic alterations of the *EIF3F* gene in the TCGA LUAD cohort of human lung tumors (1144 samples; obtained from Cbioportal). *P *<* 0.05, **P *<* 0.01, ***P *<* 0.001

To assess the impact of EIF3F ectopic overexpression on cancer biology, we generated an in vivo orthotopic mouse model of human LUAD using EIF3F-A549 cells expressing luciferase (Fig. 2a). Detection of human cancer cells in the murine lung was confirmed using anti-HLA antibodies (Fig. 2b). It can be seen that human A549 cells generate carcinomas in the murine lung parenchyma. A549 human cells invasion in the mouse lung parenchyma was also observed when EIF3F was overexpressed (Fig. 2b). Following engraftment of EIF3F-*luc*-A549 cancer cells or corresponding controls (CTL) in NOD-scid-gamma (NSG)-mice, orthotopic lung tumor growth was observed via in vivo luminescence bioimaging for 4 weeks (Figs. 2a, c). Measurement of tumor volume revealed that EIF3F ectopic overexpression significantly reduced the tumor size in vivo (Figs. 2d, e). After 4 weeks, the tumor volume was reduced by a factor of three (*p* < 0.05) when EIF3F was overexpressed in the injected A549 cells (Fig. 2e). No change in body weight was observed between the two groups of mice. At week 4, cancer cells bioimaging also revealed the presence of hepatic metastasis (Fig. 2f), which is associated with poorer progression-free survival and overall survival in stage IV LUAD patients [11]. Quantification of hepatic metastasis volume showed a significant increase in mice that initially received the EIF3F-*luc*-overexpressing A549 cancer cells during the engraftment (Fig. 2g). We verified the effect of EIF3F overexpression on metastasis in two additional orthotopic mouse models of LUAD generated with H460 and H1975 human lung cancer cells. An immuno-competent model of lung cancer cells metastasis was also generated using murine LLC cells. In the two human orthotopic LUAD models a significant reduction of tumor size was observed (Supplementary information Fig. S2A, B). A larger number of metastatic animals was also observed in the ‘H1975_LUAD mice’: 6/10 in the control H1975 group versus 10/10 in the H1975-EIF3F group (Supplementary information Fig. S2C). In the ‘H460_LUAD mouse’ model the large tumor size led to premature animal death before metastasis could be evaluated. In the LLC_LUAD immuno-competent mouse model of metastasis, we observed a significant increase in the number of hepatic tumors following EIF3F-*luc*-LLC cells intra-venous injection (Supplementary information Fig. S2D)

The link between EIF3F overexpression and increased hepatic metastasis was also explored in human samples in silico using ‘TCGA database [9]’, focusing on the cohort of patients with lung adenocarcinoma (LUAD TCGA cohort; http://cancergenome.nih.gov/.). First, the analysis of EIF3F expression in 584 human LUAD tumors (RNAseq data) revealed variable expression of this gene and the existence of a subgroup of tumors with high EIF3F expression (Fig. 2h; Supplementary information Table S2). Kaplan–Meier representation showed that lung tumors with high EIF3F expression were associated with a lower survival rate in patients (Fig. 2i). Moreover, TCGA-LUAD patients with high EIF3F expression presented a higher incidence of metastasis, as assessed using the criteria proposed by the American Joint Committee on Cancer (Fig. 2j; Supplementary information Table S2). Accordingly, the M1 population, which regroups individuals developing metastasis, was increased in the subgroup of patients with high EIF3F tumor expression (Fig. 2j; Supplementary information Table S2). Lastly, a genetic alteration study performed on 3592 cases with lung tumors (all types) from the TCGA did not reveal significant (<1%) gene amplification mechanisms (Fig. 2k), suggesting that EIF3F overexpression could occur at the level of gene transcription (Fig. 2j). These findings obtained in vivo and in silico indicate that EIF3F overexpression is associated with metastasis and raised the hypothesis that EIF3F could stimulate the molecular mechanisms of cancer cell migration and invasion.

### EIF3F overexpression remodels the proteome of lung carcinomas

**Fig. 3:**
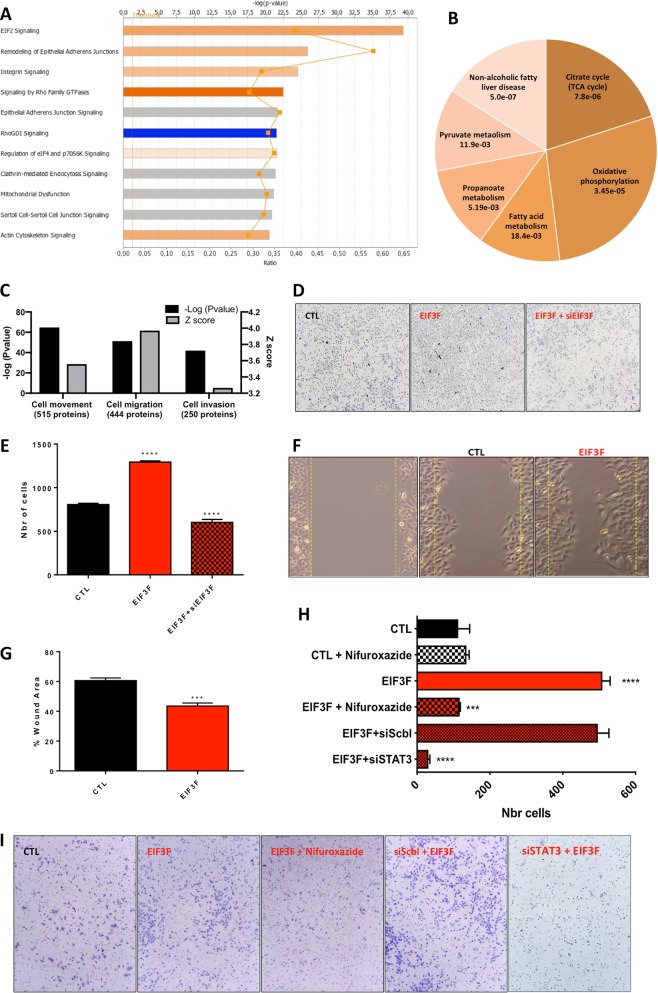
EIF3F reprograms the proteome of mice orthotopic human lung tumors and promotes cell migration. **a** Proteome analysis of EIF3F lung tumors compared to CTL lung tumors (*N* = 4). Ingenuity pathway core analysis (IPA; Qiagen). The bar graph of **a** was generated using Ingenuity Pathway analysis (IPA; Qiagen) software from the analysis of the raw data of the differential proteomics study, obtained between EIF3F-A549 and A549 orthtotopic lung tumors. The proteins with a different expression between the two types of tumors were organized based on pre-defined categories suggested by IPA (as for example ‘EIF2 signaling’). Then, proteins with different expression were assigned to these IPA-categories based on the IPA-experts curated database, and the categories were ranked according to their frequency of identification in the proteome (the –log(*p* value); top axis value). Then, the directionality of the change per category was given by a color code. Orange means that the pathway was increased, blue that it was inhibited, and gray that no directionality could be calculated (some proteins were increased but other were decreased). The ‘ratio’ given in the bottom axis indicates the % of proteins from the predetermined IPA-category that were identified in the differential proteome. For instance 62% of the proteins composing the ‘EIF2 signaling’ group were found to be differentially expressed between EIF3F-A549 and A549 cells. **b** KEGG pathways analysis of the differential proteome analysis data showing the changes in metabolic pathways induced by EIF3F overexpression in orthotopic mice tumors. These data were obtained using String (https://string-db.org/). The pie chart was obtained by plotting the number of genes in each category, expressed as percentage of the total. **c** Cellular functions impacted by EIF3F expression in the mice orthotopic human lung tumors. **d** Representative images and **e** quantification of transwell migration experiments performed in vitro on CTL-A549 cells, EIF3F-A549 cells and EIF3F-A549 cells treated with a EIF3F-siRNA (*n* = 3). **f** Representative images of CTL-A549 at *t* = 0 h plus CTL-A549 and EIF3F-A549 at *t* = 24 h. **g** Quantification of wound healing assay performed in vitro on CTL-A549 cells and EIF3F-A549 cells (*N* = 3). The area wound was measured after 24 h of proliferation in vitro. **h** and **i** Transwell migration experiments were performed in vitro on A549 cells and EIF3F-A549 cells (*N* = 6) in the presence or absence of treatment with the STAT3 inhibitor Nifuroxazide 10 µM and siRNA against STAT3. *P *<* 0.05, **P *<* 0.01, ***P *<* 0.001

First, we analyzed the impact of EIF3F ectopic overexpression on the molecular composition of mouse orthotopic human lung tumors collected after 4 weeks of growth (see Fig. 2a). The proteome of the tumors was deciphered using label-free quantitative proteome analysis and the raw data have been deposited in PRIDE [12] (see ‘Methods’). Those data were further analyzed using ingenuity pathway analysis (IPA; Qiagen) to discover potential changes in specific biological functions. First, EIF3F overexpression triggered significant changes in the content of 120 proteins (78 increased and 29 decreased; *p* < 0.05). The signaling pathways impacted by these changes are summarized in Fig. 3a; only the pathways with an enrichment score >1 (activation; orange) or <1 (inhibition; blue) are shown. The major proteomic changes occurred at the level of ‘EIF2 signaling’, which indicates modifications in protein synthesis, as expected with EIF3F ectopic overexpression. Major changes in the proteome were also observed at the level of ‘Remodeling of epithelial adherens junctions’, ‘Integrin signaling’ and ‘Epithelial adherens junction signaling’, suggesting that modifications occurred in the molecular systems involved in cell mobility, invasion and metastasis. Significant changes in the proteome were also observed at the level of ‘Mitochondrial function’, suggesting a potential impact of EIF3F overexpression on energy metabolism (Supplementary information data Table S3). Accordingly, a detailed analysis of the metabolic pathways impacted by EIF3F overexpression, using the Kyoto Encyclopedia of Genes and Genomes (KEGG), revealed significant changes at the level of oxidative phosphorylation, the TCA cycle, and fatty acid metabolism (Fig. 3b; Supplementary information Table S4). In addition to pathway enrichment analyses, the computational study of the proteome alterations identified the cellular functions impacted by EIF3F overexpression (Fig. 3c; Supplementary information Table S5). The top results indicated possible alterations in ‘cellular movement’, ‘migration of cells’ and ‘invasion of cells’ (Fig. 3c). The detail of the proteins exhibiting these functions is given in Supplementary information Table S6. Therefore, the proteomic analysis of EIF3F-overexpressing A549 cells raised the hypothesis that their cell migratory properties were modified. We tested this possibility in vitro by performing invasion (Boyden chamber) assays (Fig. 3d, e) and wound-healing assays (Fig. 3f–i). The results indicated that EIF3F ectopic overexpression stimulated cell migration and cell invasion in A549 human LUAD epithelial cells. These findings were confirmed using two additional assays of trans-endothelial migration and matrix cell invasion, respectively (Supplementary information Fig. S3A, B). The migratory effect induced by EIF3 overexpression was abrogated using a siRNA targeted against EIF3F, showing the specificity and the reversibility of this effect (Fig. 3e). Conversely, the main components of ‘EIF2 signaling’ upregulated in EIF3F-overexpressing tumors, namely ribosomal protein L3, mitogen-activated protein kinase kinase 1, eukaryotic translation initiation factor 5, ribosomal protein L31 and ribosomal protein S24 did not participate to the acquisition of the cell migratory properties induced by EIF3F overexpression (Supplementary information Fig. S3C, D). These findings indicate that EIF3F overexpression reshapes the proteome of lung carcinomas and upregulates several proteins involved in cell migration and invasion.

### EIF3F partners with nuclear transcription factors including STAT3

EIF3F was recently discovered in the nucleus of human cancer cells and was proposed to participate in DNA-related functions [7, 8, 13]. This localization is in agreement with EIF3F interactome analyses that revealed 20 EIF3F-interacting proteins located in the nucleus (Supplementary information Table S1). However, these findings were based on parcellar biochemical studies performed on different models, and they could not be directly considered for the study of EIF3F potential nuclear functions in human lung cancer cells. Therefore, we deciphered the EIF3F interactome in human A549 cells using immunopurification and mass-spectrometry-based identification of the EIF3F protein partners. For this purpose, we generated A549 cells overexpressing a Flag-M2-tagged version of EIF3F. The EIF3F-interacting proteins with the highest scores of identification (SEQUEST > 100; Supplementary information Table S7) were located in the cytosol, and included members of the EIF3 complex: EIF3A, EIF3B, EIF3C, EIF3D, and EIF3CL. The KEGG pathways analysis of the EIF3F-binding partners revealed enrichment in ‘Ribosome (top score; 29 partners)’, ‘Spliceosome’ and ‘DNA replication’. However, the results also showed that EIF3F physically associates with 254 proteins located in the nucleus and detailed analysis of the EIF3F-binding nuclear partners identified transcription factors (Table 1). First, the general transcription factor II-I (GTF2-I) and the bcl-2-associated transcription factor 1 (BCLAF1) were identified with the highest scores. Next, signal transducer and activator of transcription 3 (STAT3) was also identified, and numerous studies have revealed a key role for this transcription factor in the control of cell migratory properties and metastasis [14]. To further evaluate the role of STAT3 in controlling EIF3F-mediated cellular migration we performed knockdown and inhibition studies using siRNAs and nifuroxazide, a small molecule inhibitor of STAT3 that is approved for use in the clinic, respectively. The results of the invasion assay (Fig. 3h, i) confirmed the role of STAT3 in the acquisition of malignant invasive properties in A549 cells overexpressing EIF3F. Indeed, cell treatment with 10 µM nifuroxazide or with STAT3 siRNA showed a major decrease in EIF3F-stimulated cell invasion, while no significant effect was detected with a control (scramble) siRNA. These findings indicate that EIF3F regulates cell migration in a STAT3-dependent manner. Accordingly, immunoprecipitation (pull-down) analyses revealed the protein–protein molecular interaction between EIF3F and STAT3 (Supplementary information Fig. S4A, B). Conversely, BCLAF1 and GLF2I knockdown, two transcription factors interacting with EIF3F (Table 1) had no effect on cell migration (Supplementary information Fig. S4C, D).

**Table 1:** List of the transcription factors co-immunoprecipitated with EIF3F and identified by proteomic analysis

Accession	Description	Score	Coverage	# Peptides	MW [kDa]
P53999	Activated RNA polymerase II transcriptional coactivator p15	8.65	28.35	3	14.4
H0YF14	Bcl-2-associated transcription factor 1	5.34	7.49	2	22.1
E9PK09	Bcl-2-associated transcription factor 1	38.57	27.72	16	83.1
A0A1W2PQ43	Bcl-2-associated transcription factor 1	38.57	28.59	16	80.6
E9PJA7	Bcl-2-associated transcription factor 1	17.93	25.05	8	52.9
E9PK91	Bcl-2-associated transcription factor 1	38.57	23.08	16	100.3
E9PKI6	Bcl-2-associated transcription factor 1	38.57	26.73	16	86.3
E9PQN2	Bcl-2-associated transcription factor 1	38.57	26.80	16	86.2
Q9NYF8	Bcl-2-associated transcription factor 1	43.90	23.37	18	106.1
C9J6M0	General transcription factor II-I (Fragment)	10.13	34.91	3	11.9
P78347	General transcription factor II-I	128.42	38.58	36	112.3
H0YJB9	RNA transcription, translation and transport factor	18.08	44.96	5	14.7
G3V4C6	RNA transcription, translation and transport factor	30.73	46.63	8	22.7
K7EP08	Signal transducer and activator of transcription 3	6.20	42.86	2	10.2
P40763	Signal transducer and activator of transcription 3	31.87	22.21	11	88.0
G8JLH9	Signal transducer and activator of transcription	23.26	18.01	8	76.1
O00267	Transcription elongation factor SPT5	6.66	3.50	2	120.9
H7BYN3	Transcription factor A, mitochondrial	13.07	17.35	4	25.7
Q00059	Transcription factor A, mitochondrial	24.27	28.46	8	29.1
D6RDG3	Transcription factor BTF3 (Fragment)	4.92	47.71	3	11.8
P20290	Transcription factor BTF3	6.47	34.47	3	22.2
E5RH41	Transcription initiation factor IIE subunit beta	4.68	11.62	2	22.1
P29084	Transcription initiation factor IIE subunit beta	17.18	29.21	7	33.0
M0R0K9	Transcription intermediary factor 1-beta	15.83	21.52	5	48.4
Q13263	Transcription intermediary factor 1-beta	20.69	18.08	8	88.5

### EIF3F regulates a cluster of metastasis-promoting genes

**Fig. 4:**
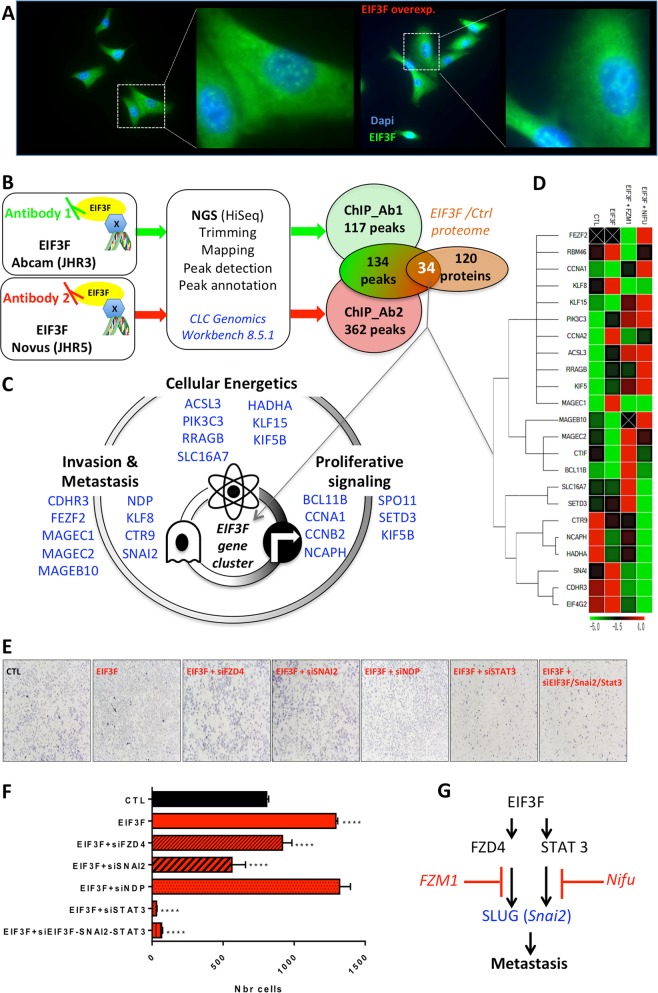
Identification of the ‘EIF3F gene cluster’. **a** Representative images of nuclear immuno-staining of EIF3F in CTL-A549 cells and EIF3F-A549 cells. **b** EIF3F gene cluster identification methods using ChIP-seq and proteomics. Following chromatin immuno-precipitation using two different antibodies, the DNA fragments associated to EIF3F were sequenced and analyzed for peak annotation. The peaks common to the two sets of experiments (134 reads) were compared to the group of proteins altered at the proteomic level (see Fig. 3) to identify the ‘EIF3F gene cluster’ composed of 34 genes. **c** Components of the EIF3F gene cluster were organized according to the Hallmarks of cancer based on their cellular function. **d** The expression of the EIF3F gene cluster components was analyzed by qPCR in A549 cells, EIF3F-A549 cells, and in A549-EIF3F cells treated with FZM1 (Frizzled-4 dependent β-catenin pathway inhibitor) and Nifuroxazide (STAT3 inhibitor) (*N* = 3). **e** Representative images, and **f** quantification of cell migration (transwell) assay performed in CTL-A549 cells and EIF3F-A549 cells using FZD4, SNAI2, NDP, and STAT3 siRNAs (*N* = 3). **g** Summary of the findings showing the implication of the EIF3F-FZD4-STAT3 signaling pathway in Snai2 expression and metastasis gene regulation. *P *<* 0.05, **P *<* 0.01, ***P *<* 0.001

To elucidate EIF3F function in the nucleus, we first verified the nuclear localization of EIF3F (Fig. 4a) and performed a chromatin immunoprecipitation experiment coupled to gene sequencing (ChIP-Seq) (Fig. 4b) using two different Chip-grade monoclonal antibodies targeted against EIF3F (Supplementary information Tables S8-1 and S8-2). The computational analysis of the DNA sequences bound to EIF3F identified 134 reads common to the two sets of experiments. The corresponding list of annotated genes was denominated ‘EIF3F-ChIP2’; it is presented in Table 2. Comparison of this list with the group of proteins with altered expression in EIF3F-overexpressing A549 cells identified 34 genes that we named the ‘EIF3F gene cluster’ (Fig. 4b, d). This cluster can be classified into three categories, according to the hallmarks of cancer [15, 16]: deregulation of cellular energetics, sustained proliferative signaling and activation of invasion and metastasis (Fig. 4c). Of particular importance for our study, the central regulator of metastasis, *Snai2* (SLUG), was found in the EIF3F gene cluster. The noncanonical β-catenin signaling activator Norrin (NDP) was also found in this list, suggesting that the Norrin/Frizzled4 (FZ4) axis could also be involved in EIF3F-mediated metastasis. To verify the extent and the directionality of transcriptional regulation of the EIF3F gene cluster by EIF3F, we performed QPCR analyses and identified 11 genes upregulated by EIF3F and six downregulated genes (Fig. 4d). Moreover, using specific inhibitors of STAT3- or FZ4-mediated transcription, namely, Nifuroxazide and FZM1, we determined the respective contribution of those pathways in the control of EIF3F-positive or EIF3F-negative gene targets (Fig. 4d). In particular, our findings revealed that the control of Snai2 (SLUG) expression by EIF3F occurs both through STAT3 and Frizzled-4-mediated transcription (Fig. 4d). The knockdown of NDP, Snai2, STAT3, or FZD4 allowed to verify their participation to the observed migratory phenotype induced by EIF3F overexpression in A549 cells (Fig. 4e, f). The results demonstrated the major role of STAT3 in the control of cell migration by EIF3F (Fig. 4f). Lastly, the expression level of proteins involved in the epithelial-mesenchymal transition (EMT) process was investigated by western blot using specific antibodies targeted against Snail, Claudin-1, E-cadherin, and Zona-occludens (ZO-1) protein (Supplementary information Fig. S5A–E). The results indicated a reduced expression of E-cadherin and an increased expression of the B-catenin, suggestive of an EMT in EIF3F-overexpressing cells. The increased level of Snail, Claudin-1, and ZO-1 also suggested that EIF3F improved the migratory phenotype of lung cancer cells, in line with the functional evaluation of cell migration shown in Fig. 2. Lastly, EIF3F-overexpressing A549 cells showed a more elongated cell morphology than the control A549s which appeared more compact (Fig. S3E, F), also suggestive of an EMT. These findings unravel the nuclear function of EIF3F in human LUAD cells and reveal the existence of a novel pathway involved in the control of cell migration (Fig. 4g).

**Table 2:** List of annotated genes bound to EIF3F and common to the two sets of ChIPSeq experiments performed using two different EIF3F antibodies

AC007682.1	EMB	PASD1
ACAP3	EMBP1	PIK3C3
ACSL3	EPHA7	PPIAL4G
ACTR3BP5	FAM230C	PROS1
AL121656.5	FAM27L	RAE1
ANKRD26P1	FEZF2	RBM46
ANKRD30BP2	FRG2	RNVU1-17
APBB2	GAREML	RNVU1-18
ARHGAP12	GYG2P1	ROCK1P1
ARL6IP6	HADHA	RPRM
ARRB1	HCN1	RRAGB
ATXN2L	ITPRIPL1	SERTM1
BCL11B	JPH3	SETD3
BRDT	KC6	SHISA9
BRSK2	KCNE4	SHROOM1
C1QTNF8	KIF5B	SLC16A7
CACNA1H	KLF15	SNAI2
CADPS	KLF8	SOWAHA
CCNA1	LINC00486	SPO11
CCNB2	LRIG3	SPRY3
CDHR3	MAGEB10	SYPL1
CRB1	MAGEC1	TGFBR3
CTIF	MAGEC2	TLL1
CTR9	MIR4429	TMEM138
CWH43	MIR4431	TMEM216
DCAF8L1	MIR4522	TMEM63C
DCUN1D4	MIR4743	TOLLIP-AS1
DDX11L5	MTRNR2L1	TPBGL
DDX18	MYO1E	TPTE
DENND1B	NCAPH	TRNAN5
DIP2C	NDP	TRNF
DPP10-AS1	NGB	TTTY23
DUX2	NPIPB9	TTTY3
EFCAB1	NPY2R	VMA21
EFHC2	NSUN7	ZMYND11
EIF4G2	NTSR2	

This list was denominated ‘EIF3F-ChIP2’. Only the characterized loci are shown

### EIF3F mediates a bioenergetic oxidative shift associated with metastasis

**Fig. 5:**
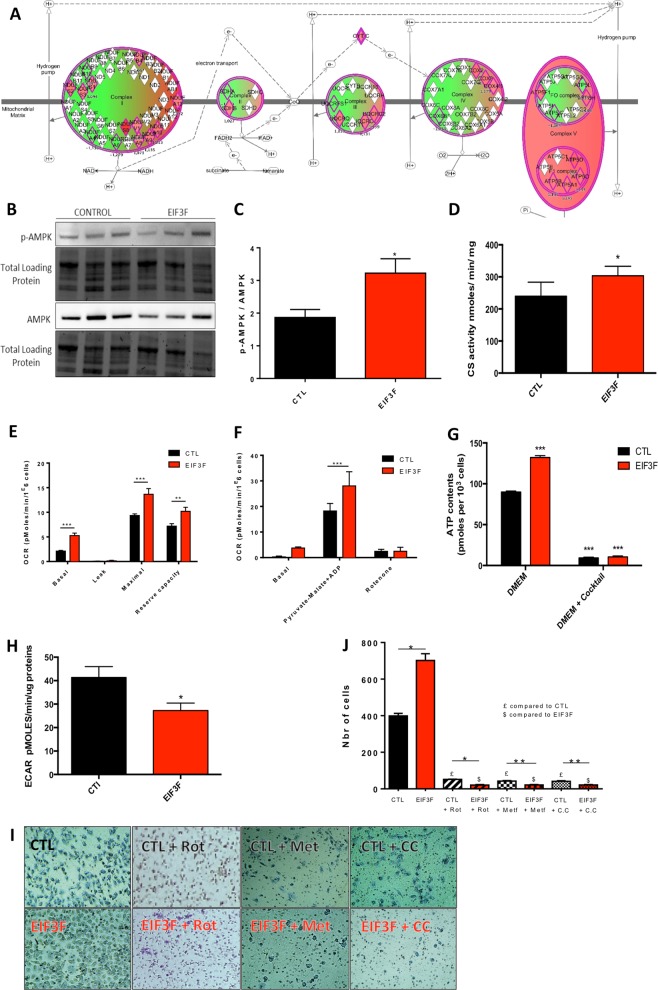
EIF3F overexpression promotes an oxidative shift in human lung adenocarcinoma A549 cells. **a** The mitochondrial proteins involved in energy metabolism and upregulated in the proteome of EIF3F-A549 mice orthotopic lung tumors are shown in red in the respiratory chain schematic representation. **b** Representative and **c** quantitative expression level of p-AMPk and total AMPK (*N* = 8). **d** Citrate synthase enzymatic activity measurement (*N* = 3) indicative of the cellular mitochondrial content in A549 and EIF3F-A549 cells. **e** Mitochondrial respiration measured in different respiratory states: basal (DMEM 1 g/L), leak (oligomycin), maximal (CCCP); see ‘Methods’. The reserve capacity was calculated as the difference between the maximal and the basal respiratory rates. The different rates were corrected for nonmitochondrial respiration determined after potassium cyanide addition (*N* = 7). **f** Mitochondrial respiration measurement in permeabilized cells (A549 and EIF3F-A549) in presence of pyruvate-malate as energy substrate. **g** Steady-state ATP content in A549 and A549-EIF3F cells. The part of ATP produced by mitochondria was inhibited using a cocktail of OXPHOS inhibitors (see ‘Methods’) (*N* = 8). **h** Extracellular acidification rate of A549 and A549-EIF3F cells (*N* = 5). **i** Quantification, and **j** representative images of cell migration (transwell) assay performed in CTL-A549 and EIF3F-A549 cells using compound C (5 μM), an AMPK inhibitor and metformin (5 mM), a complex I inhibitor (*N* = 3). For each condition the respective treated (or untreated) control was used. *P *<* 0.05, **P *<* 0.01, ***P *<* 0.001

Previous studies showed that metastasis associates with the stimulation of oxidative phosphorylation [17, 18] and that STAT3 controls mitochondrial energy production [19]. Accordingly, we found that the proteome of orthotopic human lung tumors obtained following engraftment with EIF3F-overexpressing A549 cells was enriched in 21 proteins involved in mitochondrial energy metabolism (Fig. 5a; Table S3) as F1FO-ATP-synthase subunits alpha, beta and delta or various complex I subunits. The ‘EIF3F-gene cluster’ also included effectors of OXPHOS, such as ACSL3, HADHA, or SCL16A7 (Fig. 4c). These observations suggested that EIF3F could participate in the positive regulation of genes involved in mitochondrial function. To further explore the EIF3F bioenergetic and migratory signature we studied the transcriptome of 584 LUAD patients from TCGA cohort. The tumors were first stratified based on the expression of EIF3F in the cancer tissue as compared to that determined in the noncancer tissue (Supplementary information Fig. S6A). This analysis revealed a subgroup of patients, 20% of the total population, with EIF3F expression level higher in the tumor as compared to the noncancer tissue (Z′ > 1.3-fold). Then, the expression of EIF3F-related proteins involved in oxidative phosphorylation, EMT or metastasis (as determined above) was measured in the two groups of EIF3F-high and EIF3F-low LUADs (Supplementary information Fig. S6A, B). The results indicated that mitochondrial transcription factor A (TFAM), cytochrome C1 and translocase of the outer membrane 22 were enriched in EIF3F-high tumors. A co-expression study also evidenced a positive correlation between OXPHOS proteins and EIF3F in LUAD tumors, with a higher Spearman’s correlation factor value for the respiratory chain protein subunit NDUFS3 (Supplementary information Fig. S6C, D). Likewise, markers involved in EMT, as the loss of E-caherin (CDH1) was observed in the EIF3F-high group, while activation of the N-cadherin (CDH2) was also observed in this group (Supplementary information Fig. S6A, B). Such a shift from E-caherin to N-cadherin is a recognized hallmark of EMT. Lastly, other EIF3F-linked markers of cell metastasis as CCNA1, SNAI2, KLF15, and BCL11B were also enriched in the EIF3F-high tumors. The expression data corresponding to the heatmap of Supplementary information Fig. S6 are provided in the (Supplementary information Table S9). Furthermore, enrichment study performed in the proteomics data from the group of LUAD tumors with high EIF3F expression (as selected from Fig. S6A) revealed a 4.5-fold increase in the active form of AMPK (PRKAA1_PT172), the master regulator of OXPHOS, and a tenfold increase in the AMPK target Acetyl-coA carboxylase (ACACA_PS79; see Supplementary information Table S10). Likewise, AMPK activation level was increased in EIF3F-A549 cancer cells (Fig. 5b, c) as was the activity of citrate synthase (CS), a marker of mitochondrial capacity in the cell [20] (Fig. 5d). Moreover, EIF3F-A549 cells showed an increased rate of mitochondrial respiration along with a higher reserve capacity (Fig. 5e). Bioenergetic investigations in digitonin-permeabilized cells fueled with pyruvate and malate confirmed that mitochondrial respiration was increased in EIF3F-A549 cells (Fig. 5f). Lastly, cells overexpressing EIF3F had a higher steady-state level of ATP produced by mitochondria, which was sensitive to OXPHOS inhibition (Fig. 5g). Similar findings were observed at the level of cell respiration as determined by high-resolution respirometry (Fig. S7). The rate of extracellular acidification, an indirect marker of lactate production and aerobic glycolysis, was also reduced in these cells (Fig. 5h). However, no change was observed in the steady-state level of TFAM, PGC1α, and mtDNA, as well as in mitochondrial network area (Fig. S7).

Lastly, to study the link between OXPHOS stimulation and the migratory phenotype induced by EIF3F overexpression we used the AMPK inhibitor compound C and the complex I inhibitor metformin (Fig. 5i, j). The results of the transwell assay revealed that both inhibitors altered cell migration in EIF3F-A549 cells, suggesting that OXPHOS stimulation participates to the acquisition of the cell migratory phenotype induced by EIF3F overexpression. Altogether, these findings describe an oxidative shift in the EIF3F-overexpressing A549 human lung cancer cells.

## Discussion

In the present study, we combined cell biology, genetic, and biochemistry approaches to clarify the biological function of EIF3F in human cancer cells. In particular, we deciphered EIF3F protein partners and EIF3F-binding genes, using Flagged-EIF3F-IP-proteomics and ChIPSeq, respectively. A major observation was that EIF3F associates with nuclear proteins including STAT3, a transcription factor that has been previously defined as a master regulator of cell migration and metastasis and that has been considered as a priority target for the development of anti-cancer therapeutic strategies [14]. We also discovered that EIF3F locates in the nucleus of A549 cells, binds to DNA and regulates a cluster of 34 genes (EIF3F gene cluster) involved in three major hallmarks of cancer: deregulation of cellular energetics, promotion of sustained proliferation and activation of cell migration and invasion. A key genetic finding was that EIF3F regulates the expression of *Snail2* (SLUG), a central effector of metastasis that is essential for the induction of the EMT [21]. Accordingly, we observed that EIF3F overexpression activates several molecular components involved in the EMT during proteome, western blot, and LUAD_TCGA data computational analyses. In complete agreement with our cell biology findings, we demonstrated that overexpression of EIF3F promotes metastasis in vivo using three mouse models of human or murine LUAD. While the anti-growth effect of EIF3F was important in these models, the pro-metastatic potential was weaker despite the important migratory effect observed in vitro.

The molecular mechanisms linking EIF3F overexpression and metastasis include (i) proteome remodeling with specific upregulation of the machinery involved in cell migration, invasion, and energy metabolism and (ii) nuclear activities including the partnering with STAT3 transcription factor and the regulation of a cluster of 34 genes involved in cell migration, invasion, and energy metabolism. This gene module identified in our study provides a molecular signature for EIF3F overexpression that could be used for bioinformatic studies on large datasets of tumor transcriptomes and proteomes. In particular, enrichment of this gene module in metastatic tumors could be explored. The ‘EIF3F gene cluster’ includes elements associated with metastasis involved in the control of cell migration, cell invasion, and mitochondrial energy metabolism. The control of the metastatic properties in carcinomas is very complex, and no mechanism has been described so far at the level of the EIFs [22, 23]. In addition to changes in cancer cell motility, an emerging principle of metastasis is the reprogramming of energy metabolism pathways. However, although there are previous findings suggesting that metastasis associates with increased OXPHOS [17, 24, 25], there are other data indicating that EMT is linked to reduced mitochondrial respiration [26]. Moreover, conflicting studies have been published regarding the bioenergetic phenotype of metastatic cells (glycolytic [27] or OXPHOS [17, 24, 28]), the implication of EMT in metastasis (obligatory or not [23, 29]) and the bioenergetic profile of cells undergoing EMT (glycolytic [26] or high-glycolysis combined with OXPHOS [30]). In particular, there are recent publications supporting that increased expression of EMT-linked transcription factors such as Snail and Twist causes reduced oxidative metabolism [31, 32], and that mitochondrial dysfunction induces invasive phenotype in lung cancer [33], in contrast with our findings. Accordingly, in a recent review article, Williams and Fingleton [34] wrote that ‘Various metabolic phenotypes such as aerobic glycolysis, increased glutamine consumption, and lipolysis’ have been associated with the process of metastasis’ [34]. In fact, several determinants as the oncogenic profile, composition of the serum and the metastatic niche, patients nutritive diet, microbiota composition, primary tumor localization, and tumor history in terms of treatments, age of the patient at tumor diagnosis and smoking history will impact the bioenergetic profile of cancer cells and metastatic ones. This very complex situation suggests that tumor bioenergetics could (should) be assessed for each patient using a biopsy or noninvasive imaging methods but such approaches are only beginning in the clinical setting. The work of Ralph Deberardini’s group revealed that OXPHOS is active in human lung tumors [35, 36] but no such work has yet been performed on metastasis (surgical pieces) to our knowledge, so the question remains open. Furthermore, the process of metastasis might involve cell-to-cell bioenergetic cooperative mechanisms that have also not yet been described in human tumors. We discovered that EIF3F-induced EMT markers and fosters metastasis, in association with an oxidative shift in A549 cells and in EIF3F-high human lung tumors transcriptome. We also found that AMP-activated protein kinase (AMPK), a master regulator of OXPHOS was activated in EIF3F-overexpressing tumors and in EIF3F-A549 cells. Inhibition of OXPHOS using compound C or metformin resulted in the decrease of cell migration, suggesting that mitochondrial bioenergetics participates to the migratory phenotype controlled by EIF3F. In our study we could not detect any change in the mtDNA/nDNA ratio, in the mitochondrial network area or in the expression of PGC1α and TFAM, while CS activity was increased as were mitochondrial ATP level and mitochondrial respiration. Accordingly, recent work showed that AMPK stimulation may not systematically increase mitochondrial biogenesis per se, but rather modify the turnover of the organelle leading to a higher quality control and improved efficacy/stability of the ETC proteins [37]. Still, AMPK regulates many functions of the mitochondrial machinery, and the questions linked to organelle turnover remains open.

Therefore, our findings demonstrate that EIF3F overexpression confers to cancer cells most molecular and bioenergetic attributes of metastatic cells.

The underpinning mechanisms highlight the complexity of EIF3F biological functions with a previously identified ‘translatomic’ role at the level of mRNA degradation [10, 38] and additional nuclear activities as evidenced in our study and in previous works [7]. Our analysis of the proteins co-immunoprecipitated with EIF3F revealed that besides the known cytosolic interactors of this factor, similar to other EIFs and ribosomal components, a significant number of elements were located in the nucleus. In particular, a physical association between EIF3F and transcription factors was discovered in our study. The GTF2-I showed the highest identification score, according to mass spectrometry, followed by bcl-2-associated transcription factor 1 (BCLAF1) and STAT3. GTF2-I is involved in the formation of multiprotein complexes and interacts with the basal transcription machinery, BCLAF-1 is a death-promoting transcriptional repressor that interacts with several members of the BCL-2 family of proteins, and STAT3 is a signal transducer and a transcription activator that mediates cellular responses to interleukins and other growth factors. Using knockdown approaches of GTF2-I or BCLAF-1 we showed that these two transcription factor do not participate to the regulation of cancer cells migration. Conversely, STAT3 is a well-described player of the metastatic cascade as confirmed in our work. STAT3 regulates genes involved in proliferation, apoptosis, survival, angiogenesis, invasion, and metastasis, as well as genes responsible for mitochondrial biogenesis. Interestingly, a large number of proteins upregulated in the proteome of EIF3F-overexpressing cells belong to the β-catenin signaling pathway, and recent studies revealed that STAT3 cooperates with β-catenin via direct physical interaction to promote cancer cell malignancy and chemoresistance [39].

A further characterization of the mechanisms linking EIF3F to STAT3 and β-catenin might lead to the development of novel therapeutic strategies. In particular, there is high interest in discovering small molecule inhibitors of EIFs [40], but EIF3F-specific inhibitors remain to be identified. Our work shed light on the genetic mechanisms controlling cancer cell migration and invasion, which remain the greatest challenge in the clinical management of cancer [23]. Indeed, ~50% of patients with lung cancer have metastasis in different organs [41]; thus, the 5-year survival rate for patients with metastatic lung tumors (stage 4 and above) is dramatically low at ~1%.

The discovery of a role for EIF3F in the genetic control of cell migration and metastasis in human LUAD could lead to the development of diagnosis and therapeutic strategies. For instance, we found that EIF3F cooperates with STAT3 and exploited this finding to successfully target EIF3F-overexpressing human lung cancer cells with nifuroxazide, an FDA-approved oral anti-diarrheal agent that has been identified as an inhibitor of STAT3 [42]. These findings could have implications for the treatment of a subset of human lung carcinomas that overexpress EIF3F (20% of the LUAD cohort) and that are associated with a lower patient survival and a higher rate of metastasis. Regarding diagnosis, EIF3F could be considered as a marker for metastasis, and immuno-histopathology studies on large cohorts of tumors will be required to verify this possibility. In particular, we observed that EIF3F overexpression had opposite effects on cancer cell proliferation and migration. This inverse relationship between the cancer cell proliferation rate and metastatic potential has already been described as a biological feature of colorectal metastasis [43] or melanoma metastasis [44]; however, it remains poorly studied in human lung carcinoma. The mechanisms underlying the observed dichotomy between invasion and proliferation were recently investigated in astrocytoma [45] and glioblastoma [46], and metabolic reprogramming was suggested to have a role in the ‘go or grow’ phenotypes; however, the link between bioenergetic deregulation, reduced proliferation and increased invasion potential remains poorly understood. Our findings indicate that EIF3F could be considered as a potential regulator of the ‘grow or go’ dichotomy, given the impact of EIF3F on cell proliferation, migration and energy metabolism.

To conclude, our multi-omic, bioenergetic and cell biology study provides a better understanding of EIF3F biological function in the nucleus, beyond protein translation. We provide a unique set of data demonstrating that EIF3F regulates the metastatic process by remodeling the proteome, the transcriptome and the bioenergetics of cancer cells. Using Chip-seq, we also demonstrate that EIF3F binds to and regulate the expression of a cluster of 34 metastasis-promoting genes. This discovery extends our knowledge of the molecular mechanisms of metastasis that causes 90% of deaths from cancer. Therefore, our findings suggest that EIF3F could be considered as a novel effector of cell migration and metastasis, and its molecular link with STAT3 fosters the need to pursue investigations on the role of the EIF3F–STAT3 signaling in cancer. Lastly, we demonstrate that targeting STAT3 in EIF3F-overexpressing human lung cancer cells inhibits cell invasion.

## Methods

### Cell culture and generation of EIF3F-overexpressing cells

A549, HeLa, H460, and H1975 human cells were obtained from ATCC (Bethesda, MD, USA). Cell culture was performed in DMEM 5 mM glucose (Gibco), supplemented with 10% FBS (Gibco) and penicillin–streptomycin 1× (100×; Gibco). The lentiviral vector used for human EIF3F overexpression was constructed by inserting EIF3F cDNA into pLenti vector, which contains a FLAG tag and a gene for puromycin resistance (OriGene). Lentiviral particles were produced by transient transfection of 293T cells using a calcium phosphate transfection technique. Prior to infection the medium was removed and 5 × 10^5^ cells were incubated with viral supernatants for 48 h at 37 °C in the presence of 8 μg/ml of protamine sulfate. EIF3F overexpressing cells were selected using promycin treatment (1 µg/mL) in culture medium.

### siRNA knockdown studies of EIF3F interactors

A549-EIF3F overexpressing cells were transfected with small interfering RNA (siRNA; MISSION esiRNA, SIGMA) targeted against EIF3F, STAT3, FZD4, NDP, SNAI2, BCLAF1, GLF2I, EIF5, MAP2K1, MRSP24, RP31, RPL3I, or negative control (Scrambled esiRNA) to reach a final concentration of 100 nM. FuGENE (Promega) was used as reagent of transfection following the manufacturer’s recommendations. Knockdown was effective after 48 h of treatment.

### Cellular oxygen consumption rate

Cellular oxygen consumption rate was measured on intact cells at 37 °C in a 1 ml thermostatically monitored chamber (1.0 × 10^6^ cells/ml/run) using a Clark oxygen electrode (Oroboros O2k High Resolution respirometer, Oroboros, Austria). Cellular respiration was determined under routine condition (in DMEM), or in the presence of 6 μM oligomycin (Leak respiration independent of ADP phosphorylation), or 8 μM carbonyl-cyanide m-chlorophenylhydrazone CCCP (maximal respiration obtained in the uncoupled state). The ‘reserve capacity’ corresponds to the difference between the maximal respiration (CCCP-stimulated) and the basal respiration obtained in the cell culture medium. Lastly, the cellular nonmitochondrial respiration was obtained after inhibition of the respiratory chain using potassium cyanide (KCN). In a second series of experiments, mitochondrial respiration was assessed under controlled delivery of energy substrates. Indeed, changes in cell respiration can be caused by differences in intermediate metabolism that provides the reduced equivalents NADH and FADH_2_ to the respiratory chain. To avoid this confounding factor we determined substrate-controlled mitochondrial respiration on permeabilized cells. Cell permeabilization was obtained using 0.001% digitonin and mitochondrial respiration was initiated with 10 mM pyruvate, 10 mM malate and, 100 µM ADP.

### Extracellular acidification rate (ECAR)

Medium acidification can reflect the use of glycolysis coupled to lactate dehydrogenase as the main energy-producing system by in cultured cells. Therefore, a metabolic shift toward oxidative phosphorylation typically associates with a reduced production of lactate by this system. Cells were seeded at 4 × 10^5^ cells/well in a Nine-well XF (Seahorse Extracellular Flux Analyzer) cell culture microplate (Seahorse Bioscience). ECAR was measured after 24 h of cell culture using a XF96 Analyzer (Seahorse Bioscience). Cells were equilibrated 1 h at 37 °C in DMEM cell culture medium supplemented with 5 mM glucose before baseline measurement.

### Cellular and mitochondrial ATP synthesis

The steady-state ATP content was measured by bioluminescence using the CellTiter Glo kit (Promega), following the manufacturer’s recommendations. The part of ATP produced by mitochondria was calculated as the difference between the total ATP content and the amount of ATP measured in the presence of the OXPHOS inhibitory cocktail containing 30 µM oligomycin, 5 µM rotenone and 1 mM KCN. Cells were incubated 30 min with the OXPHOS inhibitory cocktail to inhibit mitochondrial respiration and linked-ATP synthesis.

### Measurement of the enzymatic activity of CS

First, a cell homogenate was prepared as follow: 10 million cells were detached from the plate using trypsin-EDTA 0.25% solution and a cell pellet was obtained by centrifugation 10 min at 300 g. The pellet was frozen at −80 °C and thawed at room temperature. Then, mechanical cell lysis was performed using a glass/glass potter at 4 °C (ten strokes in the CS enzyme assay buffer described below). A cell homogenate was obtained and protein concentration was determined using the BCA assay (Thermofisher). The enzymatic activity of CS was assessed by monitoring the reduction of 5,5-dithiobis(2-nitrobenzoic acid) (DTNB) at 412 nm (extinction coefficient 13.6 mM^−1^ cm−^1^). It was followed in a coupled reaction with CoA and oxaloacetate. A reaction mixture of 0.2 M Tris-HCl, pH 8.0, 0.1 mM acetyl-CoA, 0.1 mM DTNB, and 5–20 µg of cell homogenate proteins was incubated at 30 °C for 5 min. The reaction was initiated by the addition of 0.5 mM oxaloacetate, and the absorbance change monitored for 5 min.

### Mitochondrial network immunostaining

Cells were seeded into Nunc Lab-Tek chamber slide at 5000 cells per chamber. Briefly, cells were fixed with 4% paraformaldehyde then permeabilized with 0.15% Triton X100 before 45 min of 10% bovine serum albumine saturation. Overnight incubation of primary antibodies anti-Tom20 (abCam) was performed before fluorescent-secondary antibody incubation. Cells were finally rinsed twice with PBS. Fresh PBS was added to the chambers to maintain cells integrity. Fluorescence microscopy was performed on a Zeiss Axio Observer microscope equipped with the Vivatome system with a 63× oil spring-loaded objective. The images were acquired using AxioVision (6D acquisition and vivatome modules). A minimum of 30 different cells obtained from three different experiments was randomly selected per experimental condition, and the analysis of mitochondrial morphology was performed using Morphostryder (Explora Nova).

### Western blotting

Total cells lysis was performed using a lysis buffer containing 1.5 mM EDTA, 50 mM Hepes pH 7.4, 150 mM NaCl, 10% (v/v) glycerol and 1% (v/v) NP40. Mitochondria enriched fractions were isolated from cultured cells using a specific buffer containing 10 mM Tris, 1 mM EDTA and 250 mM sucrose as previously described [47]. Total cellular extracts and mitochondrial fractions were loaded onto a 4–15% SDS-PAGE gel (Bio-Rad), transferred onto nitrocellulose membrane and revealed with different commercial antibodies: β-catenin Cell Signaling (8480S), Claudin-1 Cell Signaling (13255S), EIF3F abcam (ab64177), EIF3F Novus (NBP1-77997), FLAG M2 Novus (NBP1-06712), PGC1α Santa Cruz (sc-13067), SNAIL Cell Signaling (3879S), STAT3 Cell Signaling (8768S), TFAM abcam (ab131607), TOM20 Santa Cruz (sc-11021), ZO-1 Cell Signaling (8193S), and anti-βActin Sigma Aldrich (A1978). Fluorescent secondary antibodies and HRP-coupled secondary antibodies were used for revelation using respectively Odyssey instrument (LI-COR) or ChemiDoc imaging instrument (Bio-Rad). Protein expressions were normalization using standard protein expression or total loading protein (Stain-free system Bio-Rad).

### EIF3F immunoprecipitation coupled to proteomic analysis

EIF3F-A549 cells contain a FLAG tag, which can be bound to the Sigma Aldrich system Anti-Flag M2 Magnetic Beads. Beads are composed of Anti-Flag M2 monoclonal antibody attached to superparamagnetic iron. The M2 antibody binds to fusion proteins containing the FLAG peptide sequence. EIF3F proteins were isolated in a native preservative for protein–protein association in a mild buffer containing 50 mM Tris HCl, pH 7.4, with 150 mM NaCl, 1 mM EDTA, and 1%TRITON X-100. Following the manufacturer’s recommendations, EIF3F protein lysate immunoprecipitation on beads was performed over night at 4 °C with gentle shaking. Elution of EIF3F protein complex was eluated directly with sample buffer and sent to proteomic analysis.

### Immunoprecipitation

Experiments assay were realized with the Dynabeads protein A immunoprecipitation kit from Invitrogen. Dynabeads were coated respectively with 1 µg EIF3F (Abcam) or STAT3 (Abcam) antibodies. Total proteins from A549-EIF3F cells were extracted with a RIPA buffer. Immunoprecipitation experiments were realized with positive control, such as purified IgG and negative control using beads without antibody coating.

### Label-free quantitative proteomics

This analysis was performed by the proteomics core facility at University of Bordeaux (https://proteome.cgfb.u-bordeaux.fr/en). The steps of sample preparation, protein digestion and nanoliquid chromatography–tandem mass spectrometry analysis on Q Exactive were performed as previously described [48]. For protein identification, Sequest HT and Mascot 2.5 algorithms through Proteome Discoverer 1.4 Software (Thermo Fisher Scientific Inc.) were used in batch mode by searching against a merge of protein databases: *Homo sapiens* and *Mus musculus* (121 170 entries, Reference Proteome Set, release 2017_05). Databases were downloaded from http://www.uniprot.org/ website. Two missed enzyme cleavages were allowed. Mass tolerances in MS and MS/MS were set to 10 ppm and 0.02 Da. Oxidation of methionine, acetylation of lysine, and deamidation of asparagine and glutamine were searched as dynamic modifications. Carbamidomethylation on cysteine was searched as static modification. Peptide validation was performed using Percolator algorithm [49] and only ‘high confidence’ peptides were retained corresponding to a 1% false discovery rate (FDR) at peptide level. Raw LC-MS/MS data were imported in Progenesis QI (version 2.0; Nonlinear Dynamics, a Waters Company) for feature detection, alignment, and quantification. All sample features were aligned according to retention times by manually inserting up to fifty landmarks followed by automatic alignment to maximally overlay all the two-dimensional (m/z and retention time) feature maps. Singly charged ions and ions with higher charge states than six were excluded from analysis. All remaining features were used to calculate a normalization factor for each sample that corrects for experimental variation. Peptide identifications (with FDR < 1%) were imported into Progenesis. Univariate one-way analysis of variance (ANOVA) was performed within Progenesis LC–MS to calculate the protein *p*-value according to the sum of the normalized abundances across all runs. Only proteins with a *p*-value cut-off ≤ 0.05 were validated. A minimum of two peptides matched to a protein, and a ≥1.2-fold change in relative abundance between the two conditions (*N* = 4 in each group) were used as the criteria for identification as a differentially expressed protein. Noticeably, only nonconflicting features and unique peptides were considered for calculation at protein level. The mass spectrometry proteomics data have been deposited to the ProteomeXchange Consortium via the PRIDE [12] partner repository with the dataset identifier PXD010097. Proteins were clusterized according to their functions by using the KEGG analysis in the search tool for retrieval of interaction between genes and proteins (STRING) database. More global analysis of the data was performed via use of IPA (Qiagen). We used the ‘Core Analysis’ package to identify relationships, mechanisms, functions, and pathways relevant to a dataset. We also used the ‘regulators’ package to identify predicted regulators of the proteomic changes. Comparative analyses were also performed with IPA using the ‘Comparative Analysis’ module.

### Cell proliferation assay

Cells were seeded in a 24-well plate at 25 × 10^3^. After 24, 48, 72, and 96 h, cells were removed with trypsin and counted. The BrdU cell proliferation colorimetric kit (abcam) was used following the manufacturer’s instructions. Cells were seeded in a 96-well plate at 5 × 10^3^ cells per well. During the final 24 h of culture BrdU is added to wells, BrdU will be incorporated into the DNA of dividing cells. Colored reaction product is quantified using a spectrophotometer.

### Measurement of the global rate of protein synthesis

Cellular protein translation was assessed in A549 and EIF3F-A549 cells using the Click-iT^®^ HPG Alexa Fluor^®^ 488 Protein Synthesis Assay Kit (Thermofisher). It contains l-homopropargylglycine (HPG), an amino acid analog of methionine incorporated in every protein. Intensity of fluorescence is correlated with quantity of protein being under translation. Cells were seeded into Nunc Lab-Tek chamber slide at 5000 cells per chamber. Immunostaining was performed following manufacturer’s recommendations using a Zeiss Vivatome Axio Observer microscope (60× objective).

### RNA extraction and real time PCR (qPCR)

Total RNAs were extracted from cells using the Qiagen RNeasy kit (Qiagen) following the manufacturer’s recommendations. Synthesis of cDNA was performed using the DNase-treated RNA according to a High Capacity cDNA Reverse Transcription Kit from Apllied Biosystems. Gene expression analysis was performed using a MyiQ real-time PCR instrument and iQ SYBR Green supermix (BioRad).

### Mitochondrial DNA quantification

After collecting the cultured A549 cells, the total DNA was extracted using a DNeasy Blood & Tissue Kit (Qiagen) and the total RNA was extracted using an RNeasy Plus Mini Kit (Qiagen). First strand cDNA was synthesized in a 20 μl volume using 1 μg of total RNA, with 50 units of M-MuLV Reverse Transcriptase (RNase H^-^) and Oligo d(T)_16_ (Applied Biosystems). Real-time quantitative PCR for nuclear DNA gene RNA12S (nDNA) and mtDNA gene RNA16S was performed using IQ^TM^ SYBR Green Supermix (Bio-Rad) on a thermocycler CFX96 Touch Real-Time PCR system (Bio-Rad), as described previously [50]. Normalized units to the endogenous references (RPLP0 and GUSB) were calculated using the CFX Manager Software program.

### Transwell migration assay

Cells were seeded at 2 × 10^4^ were plated in the upper chamber with DMEM without FBS complementation to allow migration for 24 h at 37 °C. Normal DMEM medium with 10% FBS complementation was distributed in each well, below the chamber. Chambers were gently picked up before a brief PBS rinse and 0.05% crystal violet coloration. Cells at the bottom position of the chamber were photographed and counted with a binocular loop (Leica).

### Wound healing assay

Cells were seeded in 60 cm^2^ dish and cultured until they reached confluency. Wounds were scratched on the monolayer of cells using 200 μl pipette tips. Plates were washed with fresh medium in order to rinse non-adherent cells. After 24 h, cells were rinsed with PBS, incubated with 0.05% crystal violet for 1 h. Crystal violet coloration was removed and rinsed with water. Monolayers of cells were photographed using a Zeiss Primo Vert. The numbers of cells in between the wound was counted manually.

### Invasion assay

Cell invasion properties were analyzed on A549 cells control and overexpressing EIF3F using fluorimetric and colorimetric methods: ECMatrix Cell Invasion Assay (Millipore) and Tumor Cell Transendothelial Assay (Millipore). The experiments were performed following the manufacturer’s recommendations.

### Tumors cells engraftment and optical imaging using bioluminescent tumors

A549, H460, and H1975 tumor cells were transduced with a lentiviral construct containing the Fluc gene driven by an internal CMV promoter. A single intra-pulmonary injection of 1 × 10^6^ A549 cells overexpressing EIF3F was realized on 20 anesthetized mice. For the control group 1 × 10^6^ A549 control cells were injected to 20 mice. A single intra-pulmonary injection of 1 × 10^5^ H1975 or 5 × 10^5^ H460 controls cells and overexpressing EIF3F were realized on 10 anesthetized mice. The animals used for this experiment are NOD/LtSz-*scid* IL2Rγ^*null*^ (NSG) mice (males of 8 weeks) well characterized for their severe impairments in innate immunity [51]. Engraftment and development of lung tumors were followed during 4 weeks by non-invasive optical imaging using the photonIMAGER instrument (Biospace Lab), twice per week. Prior to imaging, the mice were anesthetized with isoflurane. Mice were injected intraperitoneally with 100 μL of d-luciferin 150 mg/kg (Promega) in PBS, and shaved around the tumor site and imaged during 15–30 min to reach steady-state luminescence. At the end of the experiment, mice were euthanized, and the liver was imaged ex vivo. The lung tumors were used for protein extract and histology. The institutional animal ethics committee of Bordeaux University approved all animal use procedures.

### LLC lung carcinoma model of metastasis

LLC cells were obtained from ATCC (Bethesda, MD, USA). LLC cells were transduced with a lentiviral construct containing the Fluc gene driven by an internal CMV promoter and selected with flow cytometry using GFP fluorescence properties of lentiviral particules. Cells were transduced with lentiviral vector containing EIF3F gene and puromycin resistance (OriGene). Transduced CTL-*luc*-LLC and EIF3F-*luc*-GFP were injected intravenously into immuno-competent syngeneic C57BL/6 mice. A single injection of 1 × 10^6^ cells overexpressing was realized on 10 anesthetized mice for each group. At the end of the experiment, mice were euthanized and livers were imaged ex vivo using the photonIMAGER instrument (Biospace Lab).

### Lung tumors immunohistochemistry analysis

Lung tissues taken from mice were fixed and embedded in paraffin and 4-μm sections were prepared. Tissue sections were dewaxed with xylene and rehydrated by passage through decreasing concentrations of ethanol (100–80%). Endogenous peroxidase activity was blocked by a 5 min incubation at room temperature with 3% H_2_O_2_ diluated in peroxydase block (Dako). Tissue sections were then permeabilized with a citrate buffer (pH 6.0) at 98 °C for 30 min. After rinsing in TBS-tween, slides were incubated with primary antibody anti- HLA class 1A, -B, and -C (abCam) for 45 min at room temperature. Tissue sections were then washed with TBS-Tween and incubated with seondary antibody using Flex system from Dako during 20 min at room temperature. Peroxidase activity was detected by incubating tissue sections for 10 min. with 3, 3′-diaminobenzidine. Tissue sections were counterstained with hemalum. Slides were scanned by using a digital slide scanner (Pannoramic Scan; 3D HISTECH Ltd, Budapest, Hungary) with a Zeiss objective (Plan Apochromat 40×; numerical aperture 0.95; ZEISS, Oberkochen, Germany) and a high-resolution color camera (CIS VCC-FC60FR19CL, 4MP, CIS Corporation, Japan). The images were read by using the Pannoramic Viewer software (3D HISTECH Ltd, Budapest, Hungary).

### Hemalun, eosin, and safran stainings

Lung tissues taken from mice were fixed and embedded in paraffin and 4-μm sections were prepared. The sections were stained with HES using standard procedures for proper lung orientation and morphological assessment. Slides were scanned by using a digital slide scanner (Pannoramic Scan; 3D HISTECH Ltd, Budapest, Hungary) with a Zeiss objective (Plan Apochromat 40×; numerical aperture 0.95; ZEISS, Oberkochen, Germany) and a high-resolution color camera (CIS VCC-FC60FR19CL, 4MP, CIS Corporation, Japan). The images were read by using the Pannoramic Viewer software (3D HISTECH Ltd, Budapest, Hungary).

### Chip-sequencing analysis

EIF3F-A549 and CTL-A549 cancer cells were grown in DMEM 5 mM glucose (Gibco), supplemented with 10% FBS (Gibco) and penicillin–streptomycin 1 × (100×; Gibco) for 24 h. We used 2.0 × 10^6^ cells for classical cell lysis with a RIPA buffer supplemented with proteases and phophatases inhibitors. The EpiQuik Tissue Chromatin Immunoprecipitation Kit provided by Epigentek was performed following the manufacturer’s recommandations guidelines. For plate coating, 3 µg of antibodies have been used: EIF3F (Novus NBP2-16299) and EIF3F (Abcam ab155475). DNA libraries were prepared using Tru seq Kit and NEB Next ultra II following New England Biolabs (manual E7645) protocol. DNA fragments of 280 pb were quality analyzed with DNA high sensitivity chip on Bioanalyzer 2100. DNA sequencing was performed by Fasteris (https://www.fasteris.com/dna/) on Illumina Next seq instrument with 2 × 75pb reads. Fastq sequences were aligned and analyzed mapped on GRCh38 human genome using CLC software.

### Statistical analysis

Results were expressed as mean ± SEM and analyzed using GraphPad Prism 8.1 software. The Mann–Whitney and one-way ANOVA tests were used to compare all datasets. Normal distribution of the data and variance similarity were verified using GraphPad Prism. Statistical significance was determined at *p* < 0.05. All experiments were performed with a minimum of *N* = 3 biological replicates and *n* = 3 technical replicates. For mice studies, sample size was determined using the methods described by Berndston et al. [52]. Pre-determined exclusion criteria included the absence of signal at the start of the experiment. *P *<* 0.05, **P *<* 0.01, ***P *<* 0.001.

## Supplementary information

Tables S1-S10

Fig.S1

Fig.S2

Fig.S3

Fig.S4

Fig.S5

Fig.S6

Fig.S7

## Data Availability

The mass spectrometry proteomics data generated in our study have been deposited to the ProteomeXchange Consortium via the PRIDE [12] partner repository with the dataset identifier PXD010097.
