# Supplementary figures and images for: Nuclear control of lung cancer cells migration, invasion and bioenergetics by eukaryotic translation initiation factor 3F
Source: Oncogene. 2019 Sep 16;39(3):617–36. doi: 10.1038/s41388-019-1009-x (PMC6962096; doi:10.1038/s41388-019-1009-x)

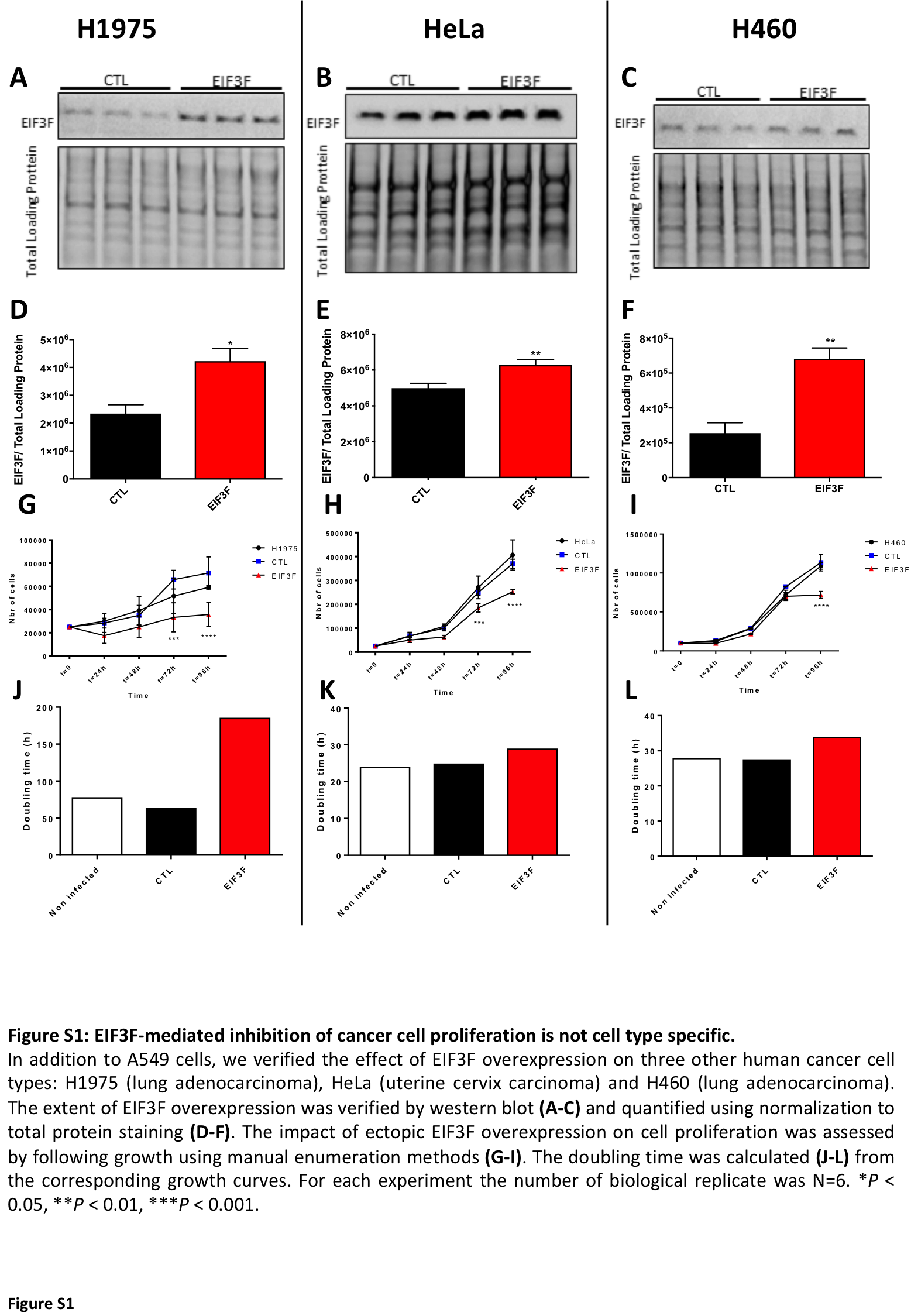

Supplement: Supplementary file 2 — Fig.S1 [file 41388_2019_1009_MOESM2_ESM.tif]

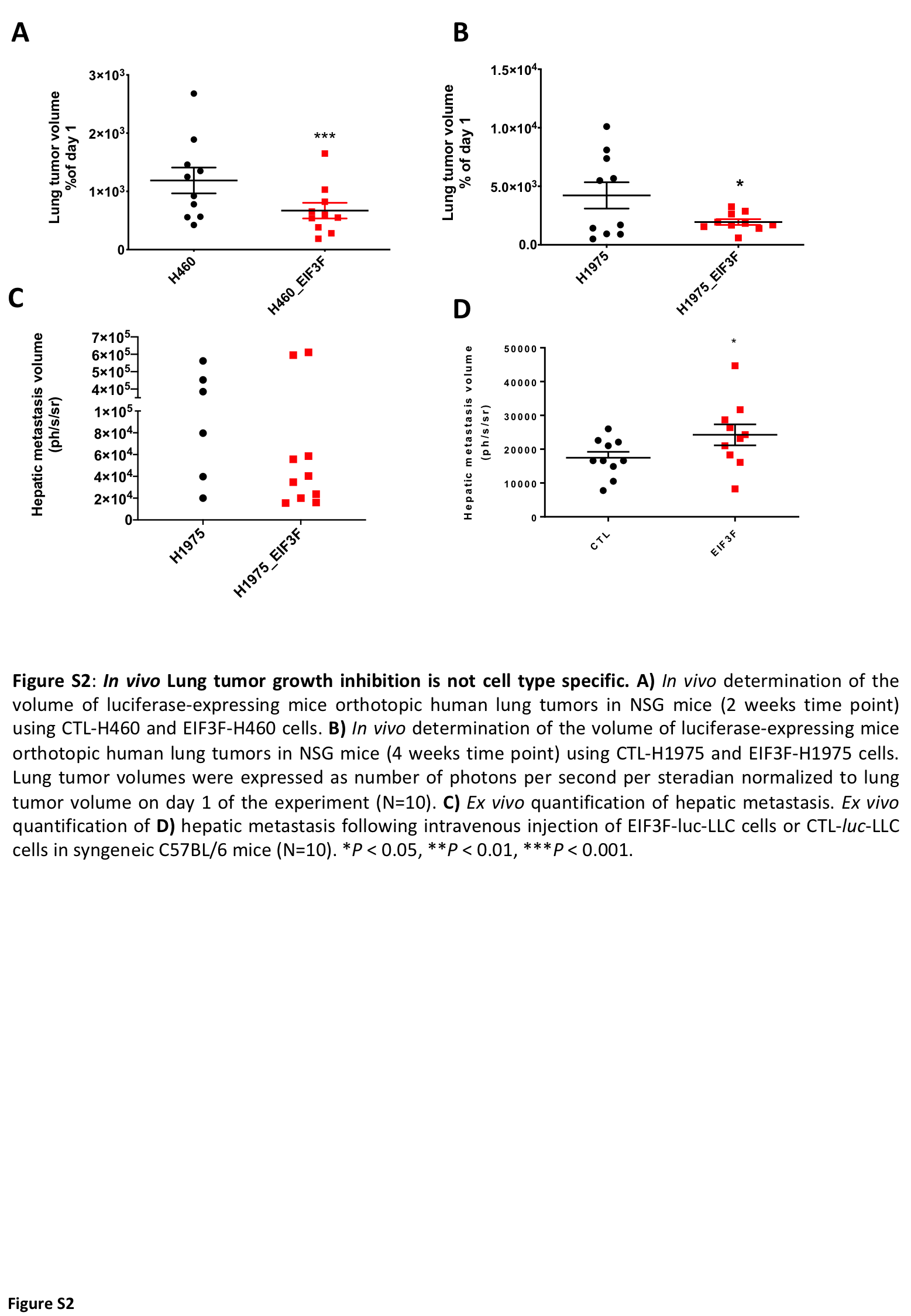

Supplement: Supplementary file 3 — Fig.S2 [file 41388_2019_1009_MOESM3_ESM.tif]

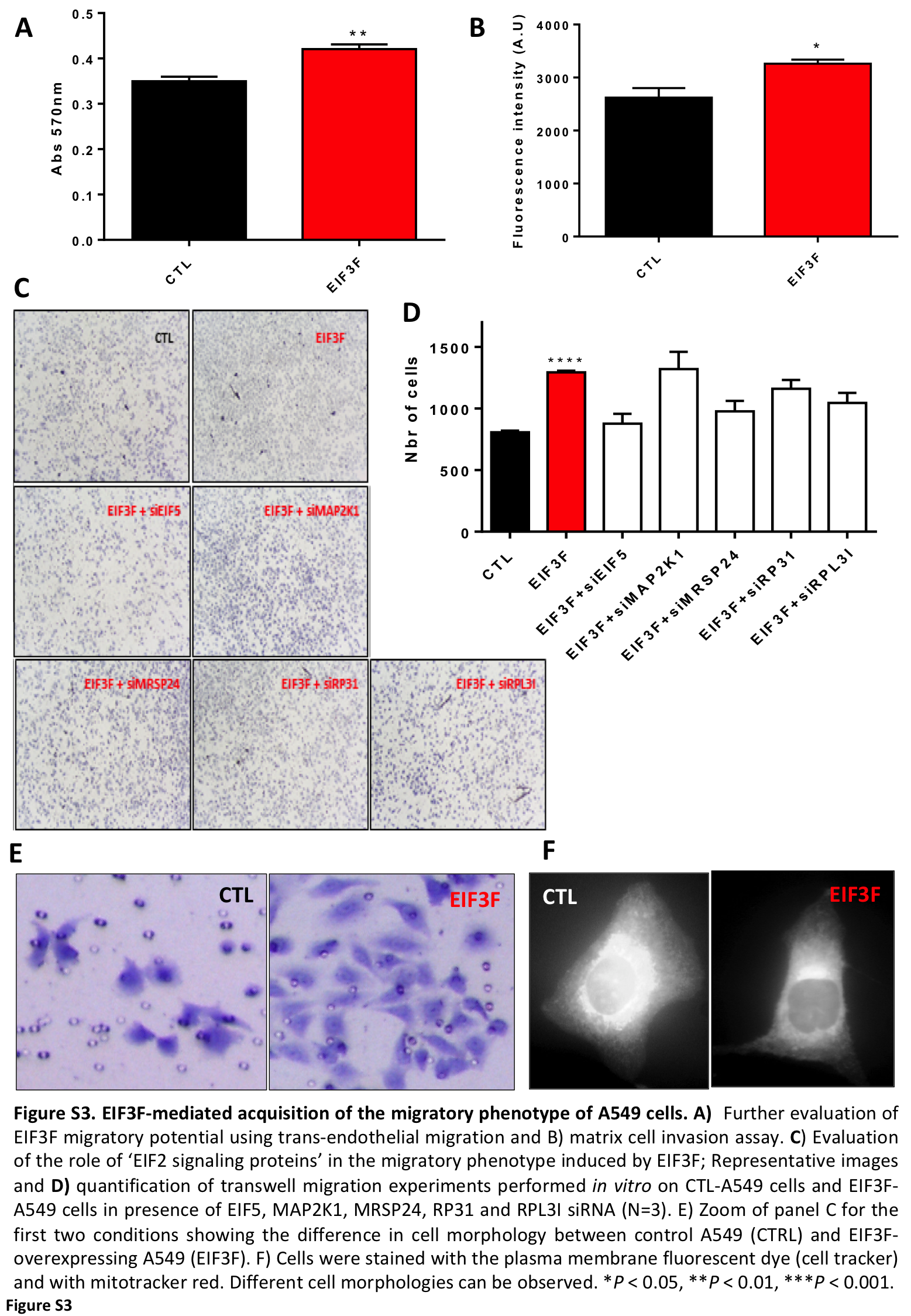

Supplement: Supplementary file 4 — Fig.S3 [file 41388_2019_1009_MOESM4_ESM.tif]

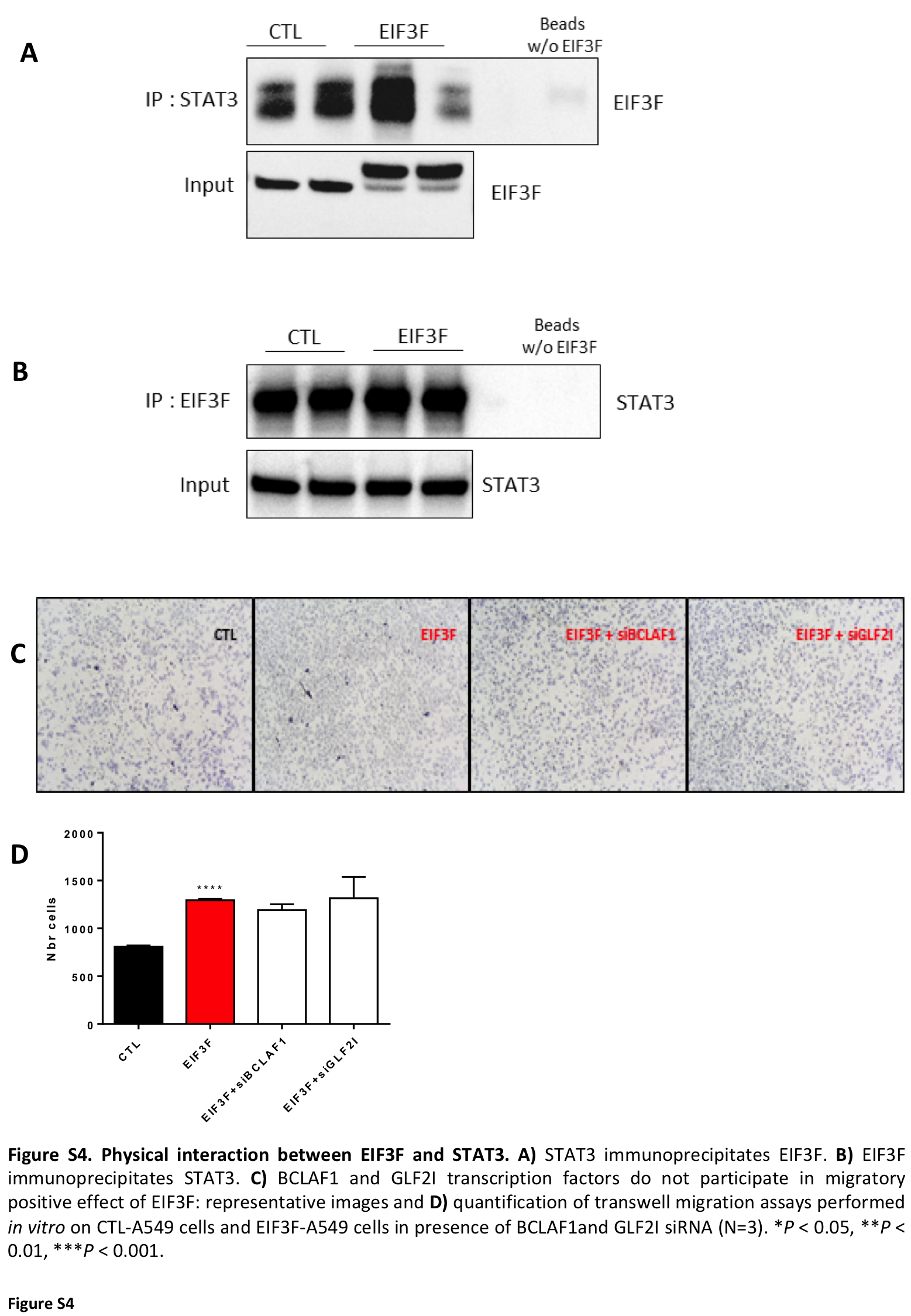

Supplement: Supplementary file 5 — Fig.S4 [file 41388_2019_1009_MOESM5_ESM.tif]

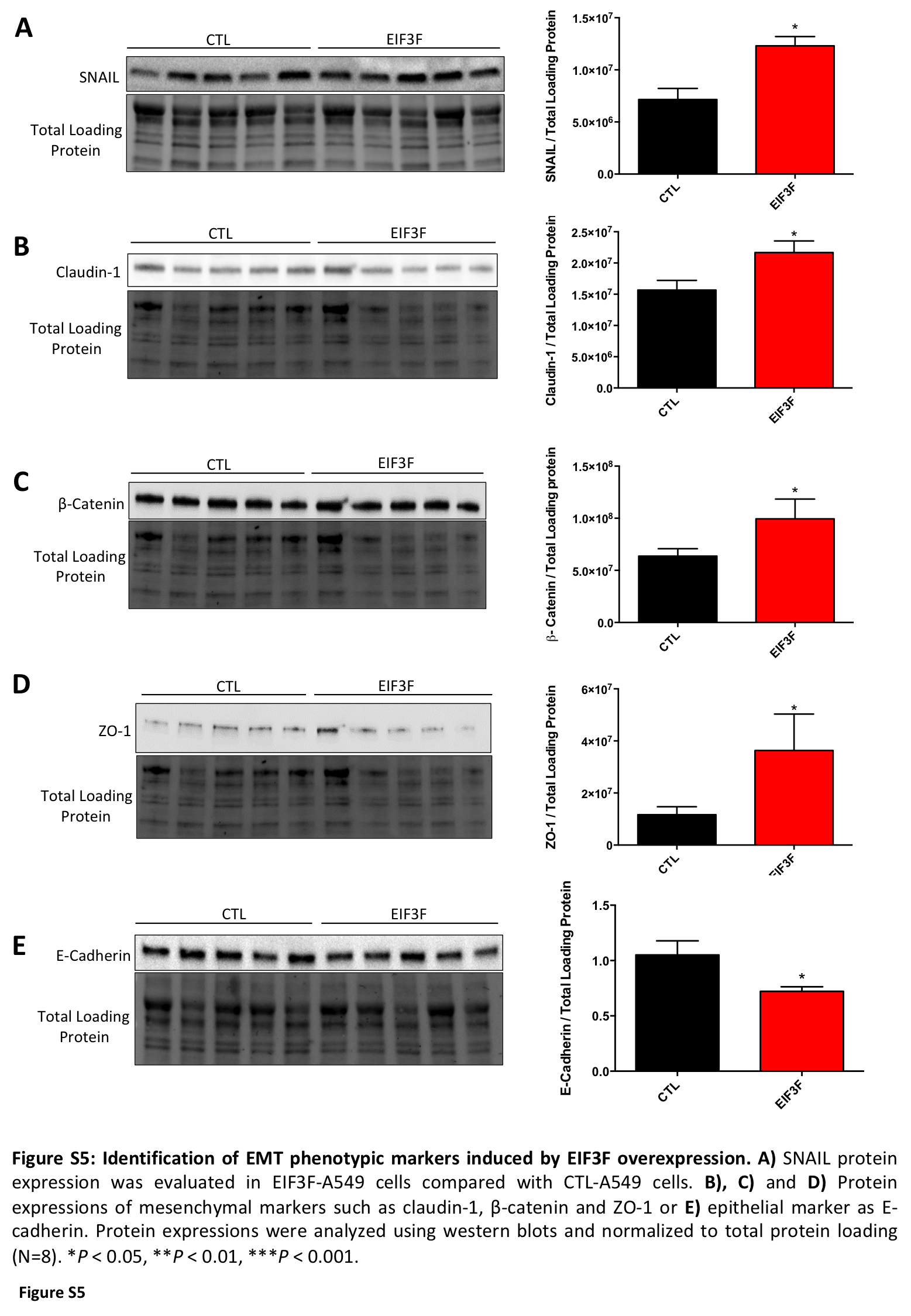

Supplement: Supplementary file 6 — Fig.S5 [file 41388_2019_1009_MOESM6_ESM.tif]

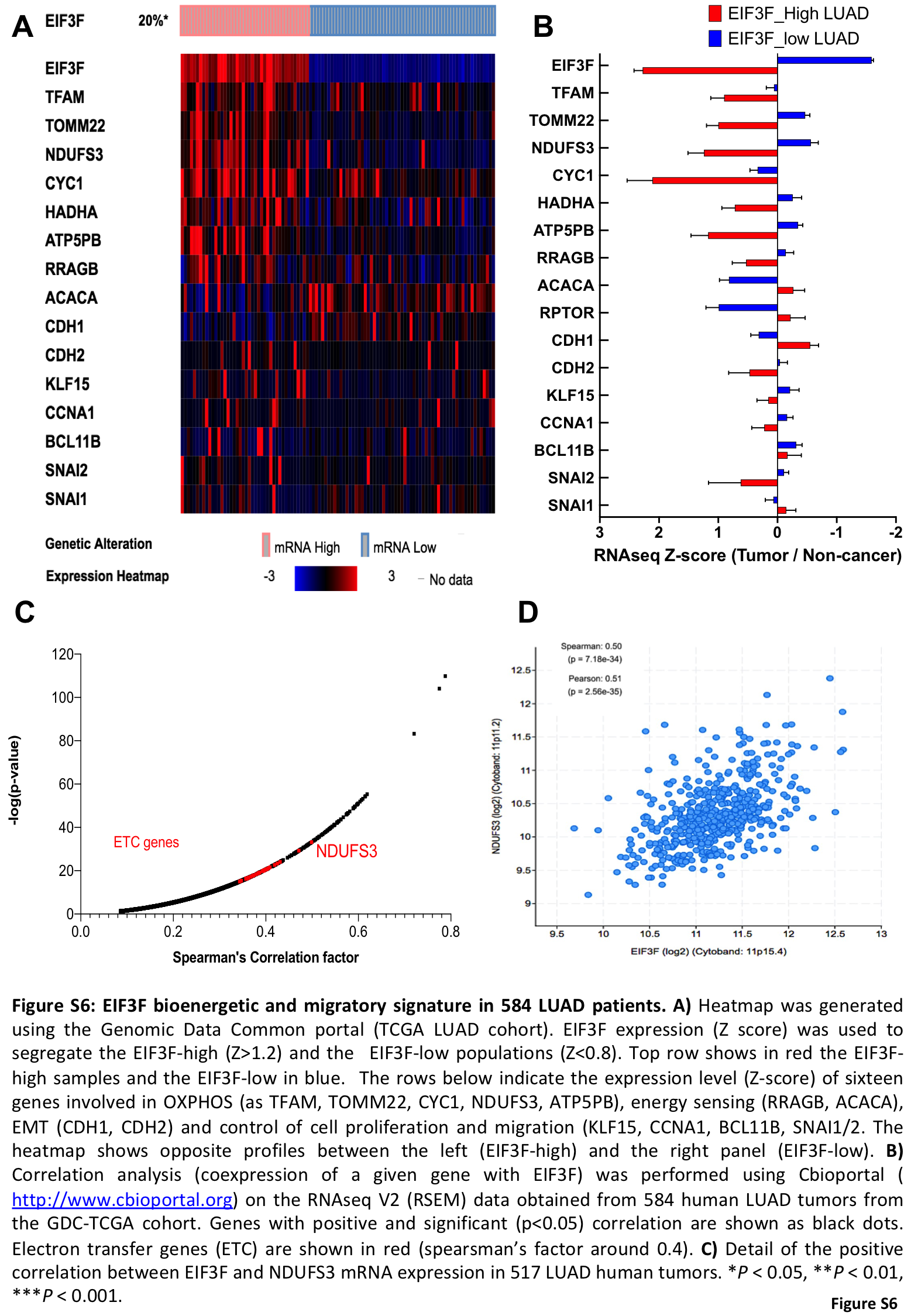

Supplement: Supplementary file 7 — Fig.S6 [file 41388_2019_1009_MOESM7_ESM.tif]

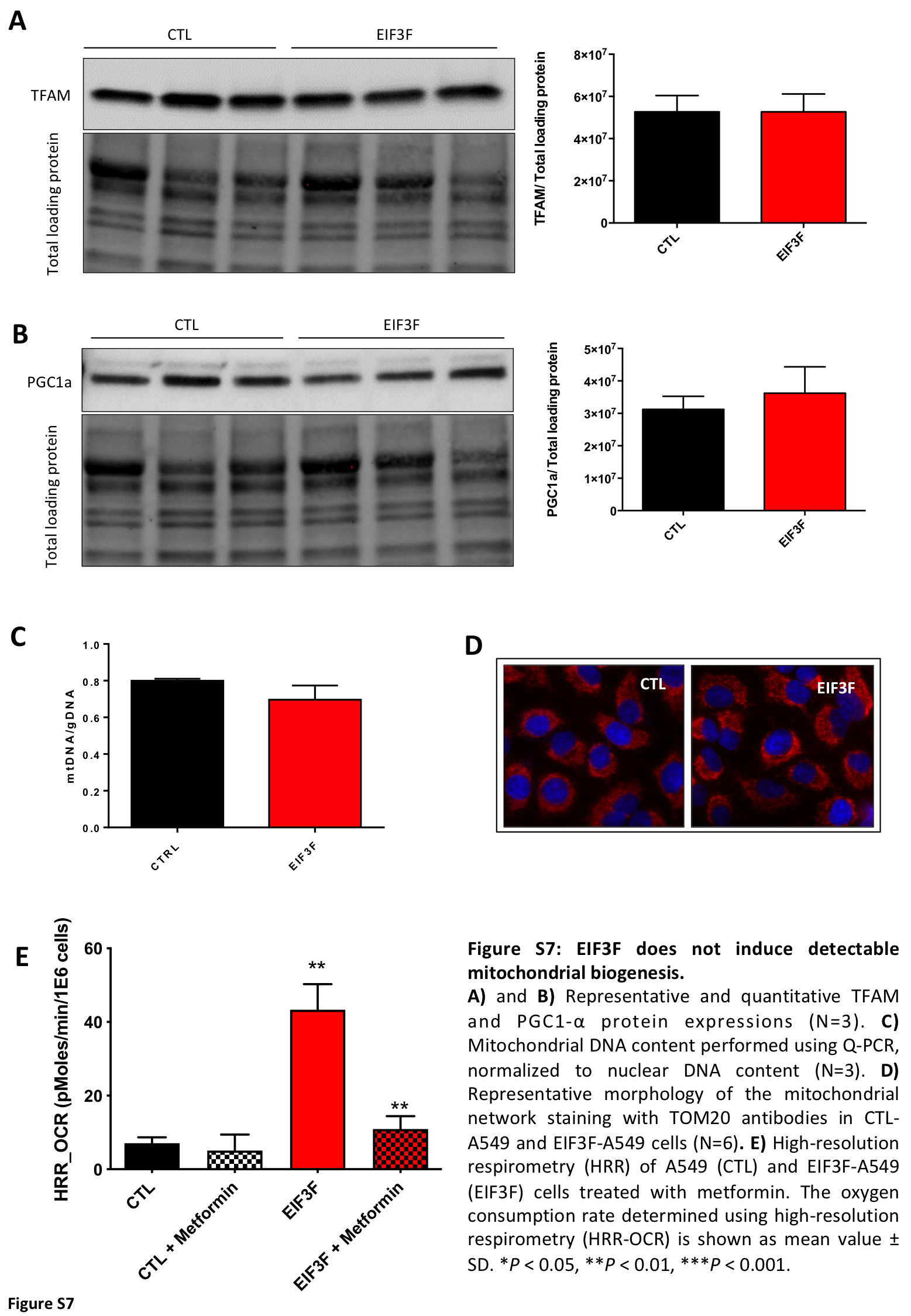

Supplement: Supplementary file 8 — Fig.S7 [file 41388_2019_1009_MOESM8_ESM.tif]
